# Noninvasive spinal neuromodulation mitigates symptoms of idiopathic overactive bladder

**DOI:** 10.1186/s42234-022-00087-x

**Published:** 2022-03-23

**Authors:** Hui Zhong, Emilie Liu, Priya Kohli, Laura Perez, V. Reggie Edgerton, David Ginsberg, Parag Gad, Evgeniy Kreydin

**Affiliations:** 1 Department of Neurobiology, University of California, Los Angeles, CA 90095 USA; 2 Rancho Research Institute, Rancho Los Amigos National Rehabilitation Center, Downey, CA 90242 USA; 3 SpineX Inc., 19509 Astor Pl, Northridge, Los Angeles, CA 91324 USA; 4 Institute of Urology, Keck School of Medicine of University of Southern California, Los Angeles, CA 90033 USA; 5 Department of Neurosurgery, University of California, Los Angeles, CA 90095 USA; 6 Brain Research Institute, University of California, Los Angeles, CA 90095 USA; 7 Institut Guttmann, Hospital de Neurorehabilitació, Institut Universitari adscrit a la Universitat Autònoma de Barcelona, 08916 Badalona, Barcelona Spain

**Keywords:** Non-invasive spinal cord stimulation, Urge urinary incontinence, Overactive bladder, Lower urinary tract, Urodynamics

## Abstract

**Background:**

Overactive bladder (OAB) affects 12 to 30% of the world’s population. The accompanying urinary urgency, frequency and incontinence can have a profound effect on quality of life, leading to depression, social isolation, avoidance of sexual activity and loss of productivity. Conservative measures such as lifestyle modification and pelvic floor physical therapy are the first line of treatment for overactive bladder. Patients who fail these may go on to take medications, undergo neuromodulation or receive injection of botulinum toxin into the bladder wall. While effective, medications have side effects and suffer from poor adherence. Neuromodulation and botulinum toxin injection are also effective but are invasive and not acceptable to some patients.

**Methods:**

We have developed a novel transcutaneous spinal cord neuromodulator (SCONE™^,^) that delivers multifrequency electrical stimulation to the spinal cord without the need for insertion or implantation of stimulating electrodes. Previously, multifrequency transcutaneous stimulation has been demonstrated to penetrate to the spinal cord and lead to motor activation of detrusor and external urethral sphincter muscles. Here, we report on eight patients with idiopathic overactive bladder, who underwent 12 weeks of SCONE™ therapy.

**Results:**

All patients reported statistically significant clinical improvement in multiple symptoms of overactive bladder, such as urinary urgency, frequency and urge incontinence. In addition, patients reported significant symptomatic improvements as captured by validated clinical surveys.

**Conclusion:**

SCONE™ therapy represents the first of its kind therapy to treat symptoms of urgency, frequency and urge urinary incontinence in patients with OAB.

**Trial registration:**

The study was listed on clinicaltrials.gov (NCT03753750).

## Introduction

Overactive bladder (OAB) is a highly prevalent condition characterized by urinary frequency, urgency and urge incontinence (Ouslander 2004; Homma et al. 2006). Although not inherently dangerous or life threatening, OAB has far-reaching implications for overall health, quality of life and health economics. Highly effective therapies for OAB exist and include pelvic floor physical therapy, oral medications, neuromodulation techniques and intravesical botulinum toxin injection (Wein 2003; Hartmann et al. 2009). Despite their effectiveness, these therapies have their respective weaknesses and shortcomings, such as low adherence, invasiveness and requirement for frequent office visits. Therefore, a need exists for additional treatment options for OAB.

The conceptual foundation for the work done here was established by our ability to restore locomotor function using lumbosacral epidural spinal stimulation (Harkema et al. 2011; Angeli et al. 2014; Grahn et al. 2017). We found that patients also began regaining voluntary control of urinary voiding (Harkema et al. 2011). In parallel, we developed epidural stimulation protocols to initiate voiding in an spinal cord injury (SCI) rat model, which normally requires manual expression of urine from the bladder (Gad et al. 2014). Our team and several others have previously demonstrated the impact of spinal neuromodulation in controlling and improving bladder function in rodents (Horst et al. 2013; Gad et al. 2014, 2016; Abud et al. 2015; Hoey et al. 2021), nonhuman primates (Havton et al. 2019) and patients with paralysis (Gad et al. 2018b; Herrity et al. 2018). More recently, we have developed and implemented a noninvasive spinal cord neuromodulation technology that delivers a sufficient electrical signal non-invasively to activate the neural structures of the spinal cord without significant cutaneous discomfort (Gerasimenko et al. 2015a, b, 2016). To date, we have demonstrated that this modality can enable recovery of voluntary movement (Gerasimenko et al. 2015b; Gad et al. 2017) of upper (Gad et al. 2018a; Inanici et al. 2018) and lower limbs (Gad et al. 2019), improved trunk function (Rath et al. 2018) and self-assisted standing (Sayenko et al. 2018). In addition to motor function, noninvasive spinal cord neuromodulation can lead to significant and meaningful levels of normalization of autonomic parameters after SCI, such as cardiovascular (Phillips et al. 2018) and lower urinary tract (LUT) function (Gad et al. 2018b), function in patients with neurogenic bladder due to stroke, spinal cord injury and multiple sclerosis (Kreydin et al. 2020; Gad et al. 2021a, b), normalize breathing and coughing after spinal cord injury (Gad et al. 2020b). Finally, SCONE™ has shown promise in improving locomotor capabilities in children with cerebral palsy (Gad et al. 2021; Edgerton et al. 2021). In our previous neurogenic bladder studies (Kreydin et al. 2020), we noted an improvement in bladder capacity and for sensate subjects - a decrease in urinary urgency and frequency. Since noninvasive spinal neuromodulation resulted in reduced symptoms of urgency, frequency and decreased incontinence episodes even in patients with a compromised nervous system, we hypothesized that similar improvement could be observed in patients suffering from idiopathic overactive bladder that have an intact neuraxis. Thus, the objective of this study was to assess the effectiveness of noninvasive spinal neuromodulation using SCONE™ in treating the symptoms of overactive bladder.

## Methods

### Patient recruitment

This study was approved by the Institutional Review Board of Rancho Research Institute, the research arm of Rancho Los Amigos National Rehabilitation Center, Downey, CA. The study was listed on clinicaltrials.gov (NCT03753750). Eight patients (7F, 1 M) were recruited for the study. The patient demographic and biometric characteristics are summarized in Table 1. The inclusion criteria included: 1) Age between 18 and 80 years and 2) Known diagnosis of overactive bladder, confirmed by either urge urinary incontinence episodes or presence of urinary frequency (> 8/day) or high urge prior to void (captured by the voiding diary). Exclusion criteria included: 1) Presence of lower urinary tract symptoms suggestive of urinary retention or obstruction, 2) Finding of an elevated post-void residual (> 100 ml) on an ultrasonographic bladder scan, 3) History of spinal cord injury, spina bifida, multiple sclerosis, stroke or other neurological disease, 4) Acute or current urinary tract infection, and 5) Current or planned pregnancy.

**Table 1:** Patient demographics

Patient ID	Age	Height (cm)	Weight (kg)	Waist (cm)	Belly (cm)	BMI
P1	53	160.0	79.5	90	96	31
P2	48	134.6	44.1	79	84	24.3
P3	69	160.0	90.5	107	114	35.2
P4	55	157.5	79.5	91	100.5	32
P5	48	165.1	90.9	122	131	33.3
P6	51	155	61.4	86	92	25.5
P7	60	142.2	46.8	89	78	33.3
P8	49	157.5	85.0	100	113	34.2

### Clinical evaluation

All patients were weaned off anticholinergic or B3 agonist medication for a period of at least 7 days prior to study initiation. All patients completed a 4-day voiding diary (2 weekdays and 2 weekend days) and all clinical surveys prior to initiation of spinal stimulation therapy and within 2 days of finishing spinal stimulation therapy. The voiding diary captured data that included: frequency of voids, frequency of incontinence episodes, urgency rating for each void (self-classified by the patient on a scale of 0 to 4, as follows: 0 = No urge, 1 = Minimal urge (Can wait 10 min or more), 2 = Moderate urge (Can wait 1 to 5 min), 3 = Strong urge (Can wait less than 1 min) and 4 = (About to leak)). If any incontinence episodes occurred, they were classified on a scale of 0 to 3, where 0 = no leak or dry pad/diaper, 1 = Small leak, 2 = Moderate leak and 3 = Heavy leak. The Minimal Clinical Important Difference (MCID) for 1) urge urinary incontinence episodes has been defined as 50% reduction, 2) urgency as 50% reduction and 3) frequency as 50% (or 8 voids or lower) by FDA’s guidance for devices to address urge urinary incontinence. The clinical surveys consisted of ICIQ-B (Cotterill et al. 2011), ICIQ-UI SF (Lim et al. 2017) and OAB-q SF (Coyne et al. 2002, 2015). At the end of the study, participants’ overall impression of symptom improvement was measured on the PGI-I scale (Peters et al. 2010), ranging from 1 (very much better) to 7 (very much worse). Skin temperature was monitored using a clinical grade infrared camera (PerfectPrime IR0280H).

### Chronic spinal neuromodulation

**Fig. 1:**
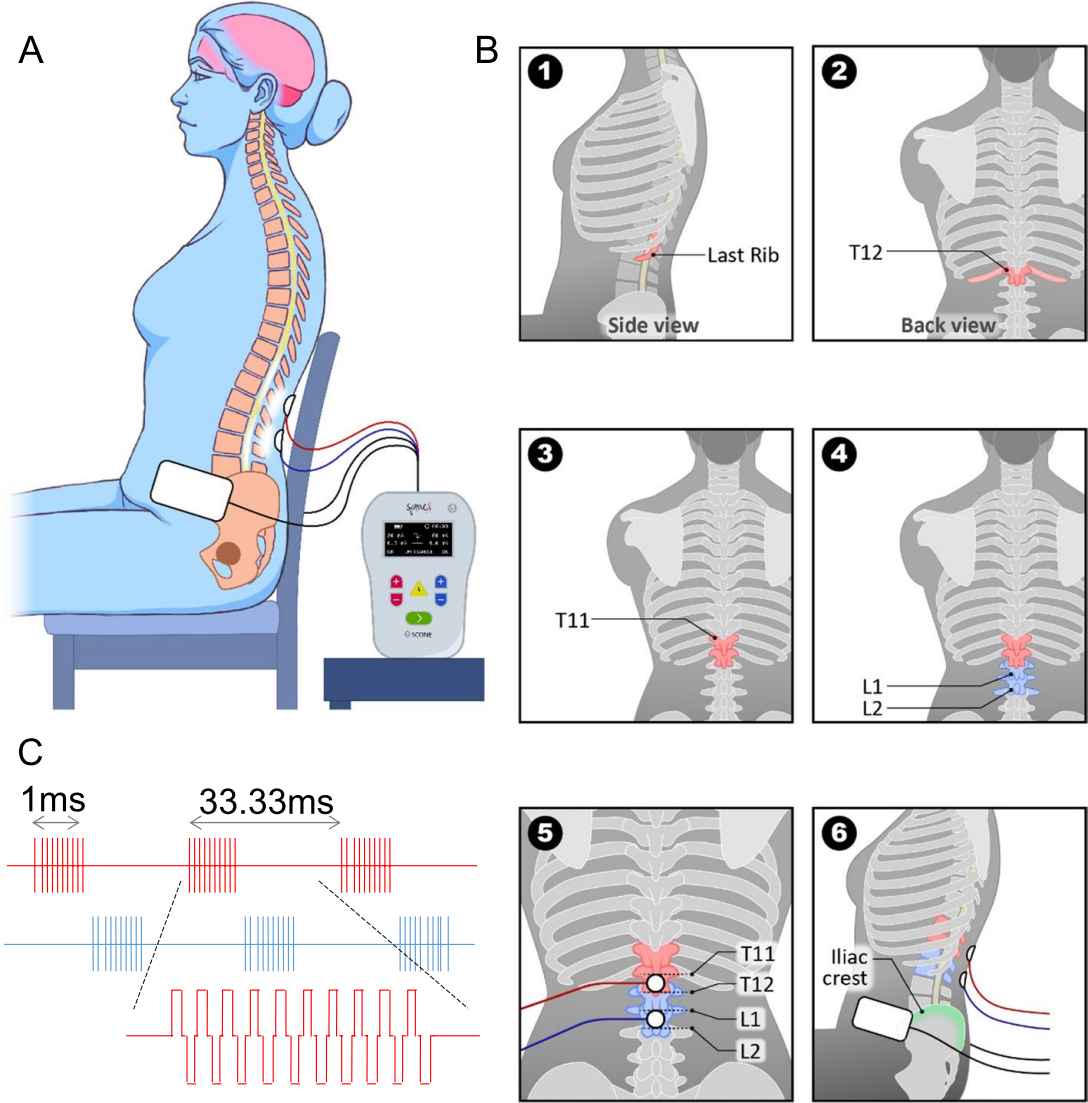
**A** Schematic representation of experimental setup. **B** Step by step representation of position the anode and cathode and **C** Electrical waveforms used in the current study

Noninvasive spinal neuromodulation was delivered using SCONE™, a proprietary electrical neuromodulation device (SpineX Inc., Los Angeles, CA). All patients completed 24 sessions at a rate of 2 sessions a week with each session lasting for 60 mins. During each session, the patients were comfortably seated in a regular office chair and were given ~ 3–5 min to ramp up the stimulation intensity and to allow for accommodation. The therapeutic waveform consisted of two alternating pulses of opposite polarities separated by a 1uS delay forming a delayed biphasic waveform (Fig. 1C). The pulses consisted of a rectangular high frequency biphasic carrier pulse (10KHz) combined with a low frequency (30 Hz) burst pulse, each with a pulse width of 1 ms. Two independent channels of stimulation were applied using self-adhesive hydrogel electrodes (1.25″ round, Axelgard) between the interspinous ligaments of T11-T12 and L1-2 serving as the cathodes and two self-adhesive rectangular hydrogel electrodes (3”× 5” rectangular electrodes, Axelgard) bilaterally over the iliac crests as the common anodes to both channels (Fig. 1). The stimulation intensity for each channel was always maintained at a suprasensory but sub-motor threshold intensity, i.e., no visible contractions were observed on abdominal, pelvic or lower extremity muscles. The intensity for each channel was adjusted individually daily based on patient comfort; i.e. amplitude of stimulation was increased until the patient could tolerate it, to identify the threshold. The stimulation was lowered to 80% of the tolerable threshold and used for the duration of the session. The intensities used across all patients ranged from 40 to 100 mA and did not change significantly over the course of the 12 weeks of therapy. Skin temperature and blood pressure and heart rate were monitored before and after stimulation. None of the patients reported any pain or discomfort due to the stimulation or otherwise during the course of the therapy.

### Statistical analysis

All data are reported as mean ± SE. Paired t-tests were used to compare the group mean data before and after stimulation. All statistical significance is reported at *P < 0.05*.

## Results

**Fig. 2:**
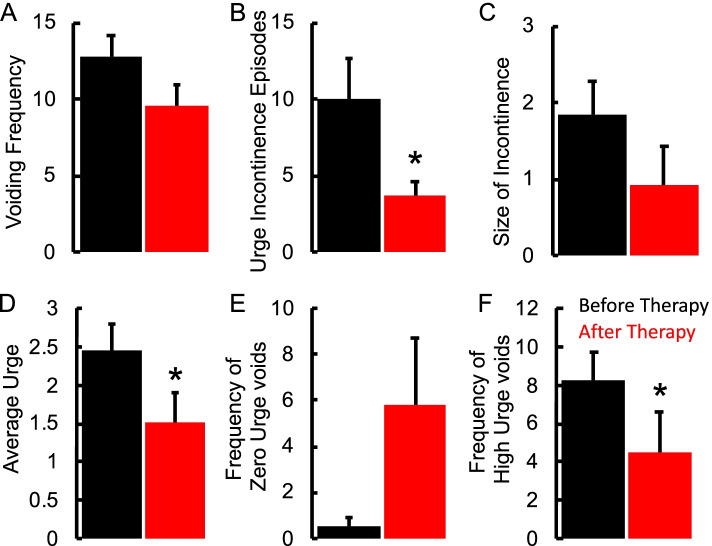
Voiding diary characteristics. **A** Mean ± SE (*n* = 8) total voiding frequency, **B** Mean ± SE (*n* = 7) urge urinary incontinence episodes per day, **C** Mean ± SE (*n* = 7) size of each incontinence episodes per day (Incontinence episodes were classified on a scale of 0 to 3, where 0 = no leak or dry pad/diaper, 1 = Small leak, 2 = Moderate leak and 3 = Heavy leak), **D** Mean ± SE (*n* = 8) Average urgency prior to voiding, **E** Mean ± SE (*n* = 8) frequency of zero urge voids and **F** Mean ± SE (*n* = 8) voiding frequency with high urge (levels 2 or higher; urgency rating for each void self-classified by the patient on a scale of 0 to 4, as follows: 0 = No urge, 1 = Minimal urge (Can wait 10 min or more), 2 = Moderate urge (Can wait 1 to 5 min), 3 = Strong urge (Can wait less than 1 min) and 4 = About to leak)

**Fig. 3:**
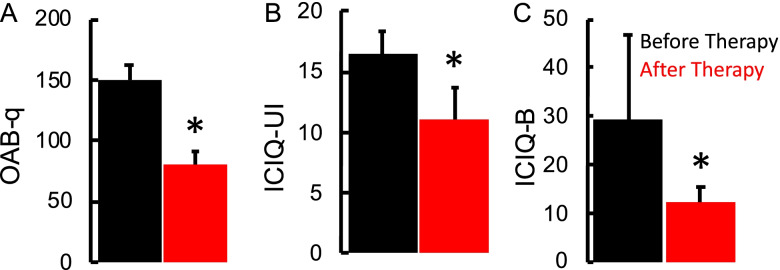
Survey scores. **A** Mean ± SE (*n* = 8) OAB-q survey, **B** Mean ± SE (*n* = 8) ICIQ-UI survey and **C** Mean ± SE (*n* = 8) ICIQ-B survey

Voiding diaries and questionnaire responses were consistent with the diagnosis of overactive bladder for all participants. Minimum daytime frequency reported was 8 voids, and minimum nighttime frequency was 3 voids at the start of therapy (Fig. 2A). Five of seven patients reported urge incontinence (Fig. 2B-C), with strong urgency (rated as 2 or greater) being common (Fig. 2D & F), occurring in 92.5% of voiding episodes across all the participants. Only 5% of voids were urge-free (Fig. 2E). Voiding diary findings were accompanied by high OAB questionnaire scores. All patients also reported high scores in their ICIQ-B surveys, suggesting concurrent bowel dysfunction. Patients were encouraged to maintain their normal lifestyle with respect to fluid consumption while in the study (Fig. 3).

Patients began to report symptom changes by week 3 of the study. In most cases, the changes observed began with improvement in urge and decrease in night-time frequency. By week 6-7, all patients also reported subjective improvement in lower urinary tract symptoms through the entire day. At week 12, all patients reported a decrease in both daytime and night-time frequency along with an overall decrease in daily voiding frequency (Fig. 2A). In addition, six out of seven patients reported improved control in the ability to plan voiding cycles with fewer high urge voids (73% at pretherapy to 45% at post therapy) and an increase in urge free voids (5% at pretherapy to 47% at post therapy) *(P = 0.11).* five out of seven patients that had urge urinary incontinence at baseline reported a > 50% decrease in the number of incontinence episodes (Fig. 2B, overall average decrease of 64%) and a decrease in the size of leaks (1.84 to 0.92, on a scale of 0 to 3) (Fig. 2C).

**Fig. 4:**
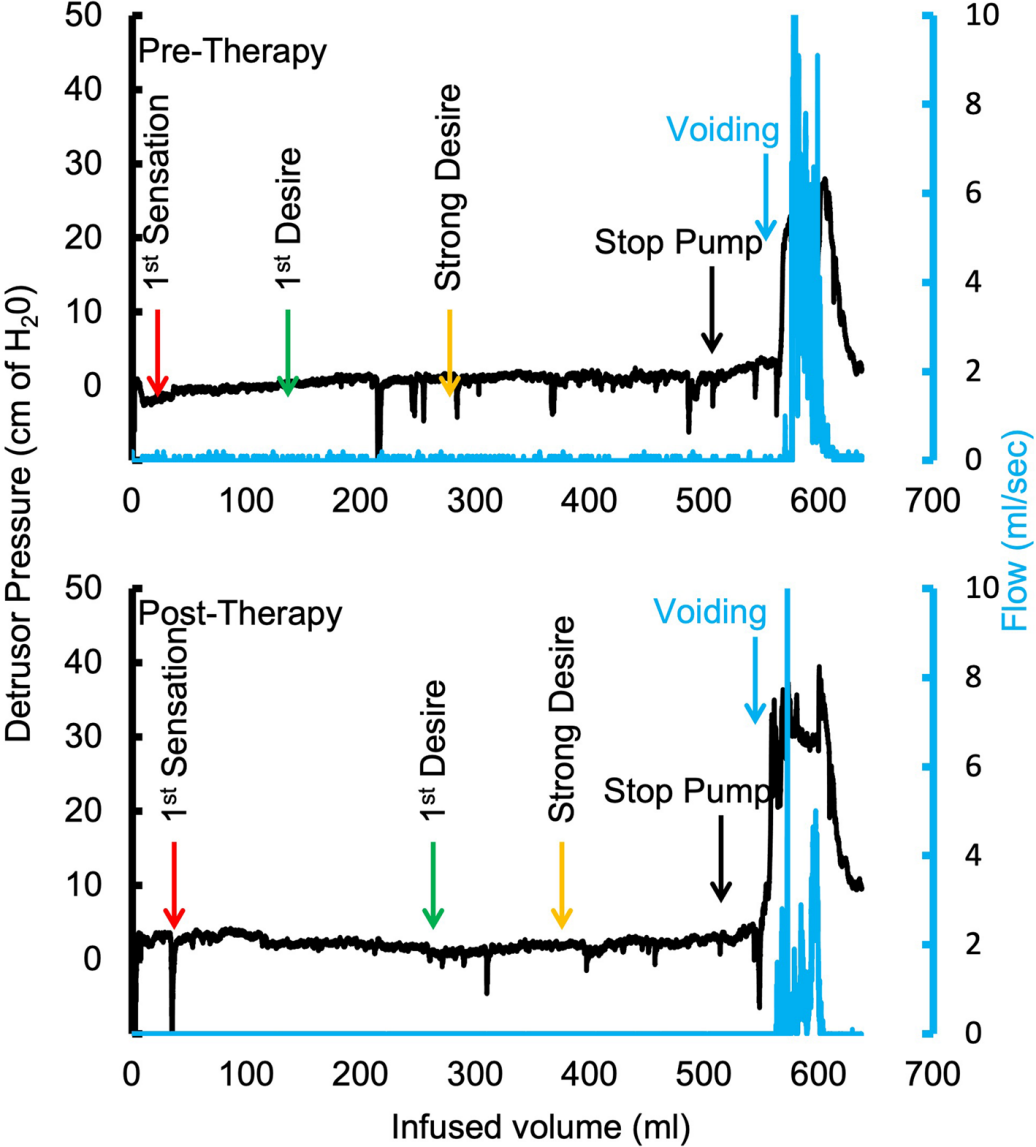
A representative Urodynamic study from a patient before and after 12 weeks of therapy. Note the while the overall bladder capacity remained the same, the changes in 1st desire and strong desire relative to beginning of voiding demonstrates the increased bladder and sphincter control

**Fig. 5:**
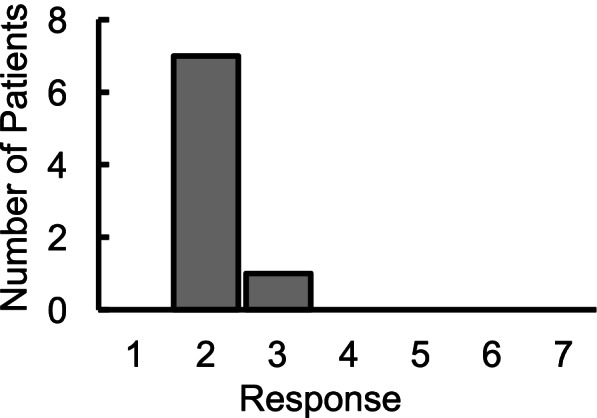
Distribution of the Patient Global Improvement Index (PGI-I), ranging from 1 (very much better) to 7 (very much worse)

Clinical questionnaire changes were consistent with the changes noted on the voiding diary, with a mean decrease in OAB-q by 72.66 ± 16.9 points and mean decrease in ICIQ-UI by 8.33 ± 2.97 points. All patients’ ICIQ-B scores also decreased by a mean of 15.5 ± 16.32 points, suggesting a significant improvement in bowel function (Fig. 3). The improvement in symptoms of overactive bladder was also reflected in urodynamic studies with patients reporting 1st desire and strong desire to void at higher volumes at post-therapy compared to pre-therapy even in the absence of active spinal neuromodulation. A representative tracing of the urodynamic study at pre-therapy and post-therapy is shown in Fig. 4. Seven patients reported to be much better on the PGI-I scale, while one reported to be a little better (Fig. 5). The average temperature did not change immediately after the stimulation was initiated and increased by < 1 °C after 60 mins of stimulation. In addition, the no significant changes in blood pressure or heart rate were observed (Table 2). No adverse events were observed, and none were reported by the patients.

**Table 2:** Changes in skin temperature and cardiovascular function before and after therapy

	Range	Pre Therapy	Stim On	Post Therapy
Skin Temperature (°F)	max	98.9	99.7	99.8
	min	98.4	99.2	99.5
Systolic (mm Hg)	max	103	Not recorded	113
	min	99		108
Diastolic (mm Hg)	max	77		82
	min	71		75
Heart Rate (BPM)	max	69		80
	min	62		72

## Discussion

In this pilot study we demonstrate that noninvasive spinal cord neuromodulation has a beneficial effect on patients with idiopathic OAB. After completing 12 weeks of therapy, patients reported improved OAB questionnaire scores, and decreased urinary frequency, urgency and incontinence. All patients reported high levels of satisfaction with the therapy. We recognize the absence of a sham arm as a weakness of the study and intend to implement a sham control in the pivotal trial that will follow these preliminary results. We also recognize that the small number of participants in the study may preclude the generalizability of the results. However, we are encouraged by the ability to detect a statistically and clinically meaningful difference in LUT symptoms even in such a small group of randomly selected participants.

Neuromodulation is a well-established therapeutic technique in OAB (Amend et al. 2011). Two main targets for neuromodulation are currently employed: the tibial nerve and the ventral ramus of the S3 spinal nerve. The S3 spinal nerve carries sensory and motor fibers that distally give rise to the pudendal nerve innervating the urethral sphincter and the pelvic nerve that innervates the detrusor muscle. Proximally, S3 fibers arise from autonomic (sacral parasympathetic nucleus) and somatic (Onuf’s nucleus) nuclei of the sacral spinal cord (de Groat et al. 2015). Thus, a possible mechanism for S3 neuromodulation is to electrically stimulate the central structures of LUT neural control in a retrograde fashion (Gill et al. 2017). Although the mechanism for tibial nerve neuromodulation is less obvious, the underlying principle is the same: the tibial nerve is partially formed by S3; thus, supra-motor threshold electrical stimulation of the tibial nerve is thought to affect S3 fibers responsible for LUT function. Like these modalities, SCONE™ appears to modulate the neural structures responsible for LUT function, with the key difference being the stimulation site: whereas existing modalities stimulate the peripheral nervous system, SCONE™ stimulation is delivered to the spinal cord, a component of the central nervous system. Whether this difference leads to any functional differences in LUT function remains to be determined; however, our early observations suggest improvement and restoration of LUT sensation as a unique property of spinal cord neuromodulation (Kreydin et al. 2020; Gad et al. 2021a, b).

In addition, we hypothesize that the mechanism of transcutaneous spinal neuromodulation is by direct stimulation of spinal cord networks involved in LUT control. By positioning the stimulating electrodes between vertebral levels T11-12 and L1-2, the electrical signal is delivered to spinal levels corresponding to the sympathetic chain and the sacral nuclei of the spinal cord respectively. We hypothesized that by stimulating these centers, SCONE would elicit a response similar to sacral and tibial nerve stimulation. The results reported here support this hypothesis with the functional improvements of OAB symptoms being similar or better compared to previously published results for these established neuromodulation modalities. Specifically, PTNS demonstrated 45% (Peters et al. 2009) to 69% (Kobashi et al. 2019) reduction in urge urinary incontinence episodes. Sacral Neuromodulation reported a 57% (Siegel et al. 2000) to 76% (Hoen et al. 2017) reduction in urge urinary incontinence episodes, whereas our current data demonstrate ~ 65% reported reduction in urge urinary incontinence episodes over the same period of time (3 months). In addition, our results demonstrate functional improvements in several other metrics extracted from the bladder diary such as the number of zero urge voids and average urgency which further strengthen our original hypothesis.

We have previously demonstrated the effect of transcutaneous spinal neuromodulation on the LUT in patients with neurogenic bladder due to spinal cord injury, stroke, and multiple sclerosis. Transcutaneous spinal neuromodulation was shown to result in improved continence, increased bladder capacity and diminished detrusor overactivity. Prior to its application in humans, spinal neuromodulation was demonstrated to affect LUT function in murine and non-human primate animal models (Gad et al. 2014; Abud et al. 2015; Chang et al. 2018). In those studies, spinal cord neuromodulation resulted in partial normalization of voiding in spinal cord injured animals (Gad et al. 2018c; Havton et al. 2019). In addition to the LUT, spinal neuromodulation has been shown to effect changes in locomotor, cardiovascular and respiratory function both in able-bodied humans and patients with neurological injuries or disease (Gerasimenko et al. 2016; Inanici et al. 2018; Gad et al. 2020a).

This study represents the initial evaluation of spinal neuromodulation for the treatment of an idiopathic condition (OAB) in a cohort of able-bodied individuals. The etiology of OAB is multifactorial, but subclinical neurological dysfunction almost certainly plays a role. Functional brain imaging studies have demonstrated that women with urge incontinence have distinct patterns of brain activation compared to women without OAB (Tadic et al. 2012). Furthermore, neuromodulation with well-established techniques, such as SNS, was shown to alter brain activity patterns in regions of the brain involved in LUT control, such as the insula and the cingulate cortex (Weissbart et al. 2018). We hypothesize that transcutaneous spinal cord neuromodulation has a similar effect on micturition-associated brain activity, potentially normalizing activation of brain regions important for LUT function.

Sacral transcutaneous electrical nerve stimulation (TENS) has been previously studied in adults (Okada et al. 1998) and is an established therapeutic technique in children, where it has been demonstrated to be effective for bowel and bladder dysfunction by multiple groups (Malm-Buatsi et al. 2007; Sillen et al. 2014; Hoffmann et al. 2018). Signal transduction to the sacral nerve in adults may not be as effective, because of the greater amount of overlying fat and subcutaneous tissue. On the other hand, dual frequency stimulation delivered by SCONE appears to penetrate the overlying tissues of the spinal cord effectively and generate an immediate and long term sensory and motor response corresponding in location to the stimulated spinal level (Kreydin et al. 2020; Gad et al. 2021b) and leading to the observed clinical changes in LUT function.

## Conclusion

This first in human study demonstrates the feasibility of mitigating symptoms of OAB using a noninvasive and well-tolerated modality, thus potentially adding a new tool to the armamentarium of OAB therapies.

## Data Availability

All raw data are available upon request.

## Abbreviations

LUT: Lower Urinary Tract
OAB: OverActive Bladder
SCI: Spinal Cord Injury
SCONE: Spinal Cord Neuromodulator
TENS: Transcutaneous Electrical Nerve Stimulation
